# Awareness, Knowledge, and Coverage of Vaccination Against Tetanus, Diphtheria, and Pertussis Among Medical Students of Karachi: A Cross-sectional Analysis

**DOI:** 10.7759/cureus.4472

**Published:** 2019-04-16

**Authors:** Amna A Siddiqui, Meeshal Khan, Jehanzeb A Khan, Syed Saad Haseeb, Aleena Mohib, Hasina M Kadri

**Affiliations:** 1 Internal Medicine, Dow University of Health Sciences, Karachi, PAK; 2 Medicine, Civil Hospital Karachi, Dow University of Health Sciences, Karachi , PAK; 3 Internal Medicine, Civil Hospital Karachi, Dow University of Health Sciences, Karachi, PAK; 4 Medicine, Civil Hospital Karachi, Dow University of Health Sciences, Karachi, PAK; 5 Community Medicine, Dow University of Health Sciences, Karachi, PAK

**Keywords:** immunization, diphtheria, pertussis, tetanus

## Abstract

Background

Along with reducing the global burden of diphtheria, pertussis and tetanus, the DPT3 (diphtheria-pertussis-tetanus) vaccine protects health care professionals (HCPs) as well as vulnerable patients in their care. This study evaluates awareness, knowledge and coverage of DPT3 vaccine and boosters among medical students of public university in Karachi, Pakistan.

Methods

A cross-sectional study consisting of 281 participants selected through convenience sampling was conducted from July 2017 to July 2018 using a self-administered questionnaire.

Results

While 93% participants received childhood doses of DPT3, only 39.2% received adult boosters. Students with positive immunization history in childhood and family vaccination routines were more likely to get adult boosters. Eighty-six percent of the students were unaware that their university had a recommended vaccination program as a part of the admission process. The assessment of awareness and knowledge revealed that only 10.5% individuals could be regarded as well-aware, 20.3% (n = 58) students as unaware, and 69.2% (n = 198) participants as moderately aware.

Conclusion

The level of awareness, knowledge and coverage of DPT3 vaccine is insufficient among medical students of a public university of Karachi; universities are recommended to take measures to enhance knowledge and ensure strict adherence of students to appropriate vaccination programs.

## Introduction

Vaccines have proved to be an ultimate tool for primary healthcare and universal public welfare. With eradication of a number of communicable diseases, it is inarguably prudent for all countries to aim and attain maximum coverage for the benefit of their population. One such vaccination is the combination DPT3 (diphtheria-pertussis-tetanus) vaccination against three common infectious diseases: tetanus, pertussis and diphtheria. According to statistics gathered by World Health Organization (WHO) in 2016, there is an 86% coverage of DPT3 vaccine worldwide [1].

Tetanus is a neurological infection caused by an anaerobic bacteria Clostridium tetani, which can be fatal, especially when present in neonates. This infection is preventable through the administration of tetanus toxoid (TT) which is available in different forms like DPT3, DT (diphtheria and tetanus), TdaP (tetanus, diphtheria and acellular pertussis) etc.Today, tetanus persists almost exclusively in the developing world, with high overall fatality rates from tetanus in adults [2, 3]. However, it has also been reported that most of the cases of tetanus in the developing world, including Pakistan, are of neonatal tetanus [4]. The reduction of tetanus in the developed world is attributed to effective vaccination programs, improved wound care and hygienic birth practices [5].

Pertussis is a highly contagious disease of the respiratory tract caused by Bordetella pertussis, a bacterium that lives in the mouth, nose, and throat. It can also be prevented by administration of inactivated whole cell (wP) or acellular (aP) pertussis vaccine, given by three primary doses via the intramuscular route, combined with diphtheria and tetanus toxoid. A study found the pertussis vaccine to be moderately effective in people aged 11 years or older, including a pertussis outbreak during the study, where the risk was reduced by 53% in participants having pertussis compared to controls [6]. In recent times, however, this preventable disease has shown a resurgence, attributed to multiple factors, including waning of protection from the vaccine [7].

Diphtheria is caused by the bacterium Corynebacterium diphtheria, and can be transmitted from person to person through close physical and respiratory contact. It can cause infection of the nasopharynx, which may lead to breathing difficulties and death. Prevention of Diphtheria can be achieved by administration of its toxoid as DPT3, DT, or Td (tetanus and diphtheria), given as at least three primary doses via the intramuscular route [8]. One booster dose of Td is required every 10 years after an initial dose of primary vaccination [9]. A Korean study concluded that the Td vaccine was sufficient in immunogenicity, even in those adults who had not had primary vaccination against diphtheria and tetanus [10].

All three of these diseases, i.e. diphtheria, tetanus, and pertussis, can be prevented with proper immunization with DTaP (diphtheria, tetanus and acellular pertussis) in children under the age of seven years, or TdaP for adolescents and adults. Immunization has a special role to play in the lives and profession of health care professionals (HCPs) and medical students. Vaccination against communicable diseases such as diphtheria, pertussis and tetanus is essential not only to safeguard the health care professional in an at-risk environment of a health care facility but also to shield vulnerable patients against carriers of these diseases, as they can serve as common reservoirs of infection for pregnant women and infants, contributing to the spread of such diseases [11]. Our study evaluates the status of awareness and knowledge regarding DPT3 immunization as well as the coverage of boosters among medical students in a public-sector university of Karachi, Pakistan.

## Materials and methods

This cross-sectional study was conducted to assess the knowledge and awareness of the medical students in a public medical college of Karachi, Pakistan towards vaccination for DPT3 and to assess the number of students who were covered by these vaccines. It was carried out from July 2017 to July 2018 after approval from the Institutional Review Board of Dow University of Health Sciences.

Participants were selected using convenience sampling from second to fifth year of study from Dow Medical College. A structured questionnaire was designed after thorough literature search, while questions were adapted and modified from previous published studies as per the requirements, and the relevant questions were added. The questionnaire was thoroughly reviewed by two proficient doctors and tested on 15 students for relevance, coherence and clarity before being self-administered to the remaining students. The sample size was calculated in open EPI Info software (http://www.cdc.gov/epiinfo/index.html) sample size calculator with a 90% confidence interval and 5% margin of error. A total of 281 medical students were enrolled in the study.

The language of instruction at this public medical college is English; therefore, the questionnaire was given in English. Before the study, the objectives of the study were explained to the students and they were informed that their participation is voluntary and informed written consent was obtained from all individuals who participated in the study. The questionnaire had two parts. The first part was about the demographics inquiring the year of study, whether they had travelled abroad recently or were international students. For our assessment of awareness regarding DPT3 immunization, students with prior travelling history or a status of an international student were excluded due to the likelihood of them having prior knowledge. The second part of the questionnaire was related to knowledge regarding DPT3 vaccine. General questions were asked about the mode of transmission of the diseases in question, vaccination schedule, booster doses and the source of their knowledge. Vaccination status was assessed by inquiring if the students were vaccinated, had received booster doses, had a family vaccination program, if their college had compulsory vaccination requirements and how these requirements were being enforced.

An Awareness Scale was drawn to assess the level of awareness and knowledge about DPT3 vaccine among the participants of this study. Eight questions were included in this scale. The first three questions were about the mode of transmission of each disease, i.e.: diphtheria, pertussis and tetanus. The possible responses were: two modes for the transmission for each diphtheria and pertussis and one response for tetanus. The next two questions inquired about the doses of DPT3 after birth and interval between the boosters. The sixth question asked what, if any, vaccination should be done after bruising due to a fall. The last two questions assessed weather the participant could appreciate that an apparently healthy individual could be infected and could identify all three listed complications of not getting vaccinated. Each correct response was given one point and the total score then graded into three groups: one-six out of 12 correctly answered questions were categorized as unaware, seven-nine out of 12 as moderately aware and 10-12 out of 12 as well aware.

Data was analysed descriptively using IBM Statistical Package for the Social Sciences (SPSS) version 23.0 (IBM SPSS Statistics for Windows, Armonk, NY) and tables were constructed using Microsoft Excel 2016 (Microsoft Corp., Redmond, WA). Chi square was used to assess the difference in the awareness related to the DPT3 vaccine between the male and female students, between medical students in their last two years of college and the second- and third-year students, and between students who had a family vaccination plan versus those who did not. Comparison of awareness was also made between the students who had been vaccinated and those who weren’t. Results for qualitative variable were presented as frequencies and percentages whereas results for continuous data were presented as mean and standard deviation. P-value was considered significant if less than 0.05.

## Results

A total of 286 students of Dow Medical College were enrolled in the study. 0.7% (n = 2) of the population was enrolled in their second year, 24.5% (n = 70) in third year, 58.7% (n = 168) in fourth year and 16.1% (n = 46) in their final, i.e. fifth year of study. Mean age of the participants was 22.21 years (SD = 1.036) and over two-third of the population was female (n = 202) with only 29.4% (n = 84) males.

Table 1 shows the knowledge of the participants regarding the DPT3 vaccination. 47.2% (n = 135) of the participants correctly answered the number of DPT3 shots required in childhood, while only 27.3% (n = 78) knew the correct time interval between the booster doses required during adulthood. Almost all the participants (98.6%) correctly identified that the tetanus vaccine is required after receiving bruises due to falling on the road. 64.7% (n = 185) and 44.4% (n = 127) of the participants recognized the mode of transmission of diphtheria being through the aerosol route and nasal discharge contact, respectively. Regarding the mode of transmission of pertussis, 90.6% (n = 259) and 40.6% (n = 116) of the participants correctly recognized them as aerosol and nasal discharge contact, respectively. Almost all of the participants (93.4%) knew the mode of transmission of tetanus correctly. 59.4% (n = 170) of participants recognized death as a consequence of not getting vaccinated, while over half of the participants reported physical impairment (60.5%) and multi-system disease (60.1%) as the consequences.

**Table 1:** Knowledge about DPT3 vaccine DPT3: Diphtheria, pertussis and tetanus vaccine; TDaP: Tetanus, diphtheria, acellular pertussis; N: Number.

Characteristics	N (%)
No. of shots in childhood	
Five shots (correct)	135 (47.2)
Others (incorrect)	151 (52.8)
Interval between adult boosters of TDaP	
10 years (correct)	78 (27.3)
Others (incorrect)	208 (72.7)
What shot to get after bruises due to falling on road?	
Tetanus (correct)	282 (98.6)
Others (incorrect)	4 (1.4)
Can a healthy-looking individual be infected?	
Yes	241 (84.3)
No	45 (15.7)
Mode of transmission Diphtheria (Aerosol)	
Recognized correct mode of transmission	185 (64.7)
Did not recognize mode of transmission	101 (35.3)
Mode of transmission Diphtheria (Nasal discharge contact)	
Recognized correct mode of transmission	127 (44.4)
Did not recognize mode of transmission	159 (55.6)
Mode of transmission Pertussis (Aerosol)	
Recognized correct mode of transmission	259 (90.6)
Did not recognize mode of transmission	27 (9.4)
Mode of transmission Pertussis (Nasal discharge contact)	
Recognized correct mode of transmission	116 (40.6)
Did not recognize mode of transmission	170 (59.4)
Mode of transmission Tetanus (Cuts, wounds)	
Recognized correct mode of transmission	267 (93.4)
Did not recognize mode of transmission	19 (6.6)
Consequences of not getting vaccinated (death)	
Yes	170 (59.4)
No	116 (40.6)
Consequences of not getting vaccinated (physical impairment)	
Yes	173 (60.5)
No	113 (39.5)
Consequences of not getting vaccinated (multi-system disease)	
Yes	172 (60.1)
No	114 (39.9)

**Figure 1:**
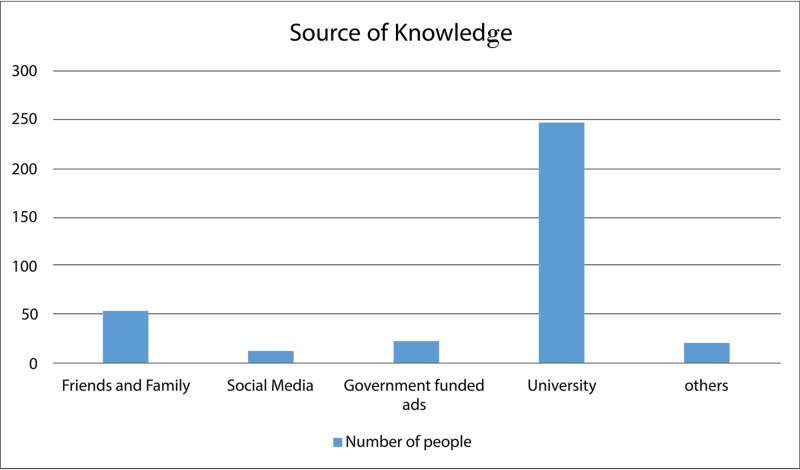
The sources of knowledge of students regarding DPT3 vaccine DPT3: Diphtheria, pertussis and tetanus

Figure 1 shows various sources of this knowledge regarding DPT3 vaccine, in which the most common source reported was the university (n = 247), followed by friends and family (n = 54), government funded ads (n = 23), other sources (n = 21) and lastly social media (n = 13).

Despite over a third (35.7%) of the population stating that their families did not have a regular vaccination routine, 93.0% (n = 266) of the participants had received DPT3 shots during their childhood. Less than half of the population (39.2%), however, had received adult boosters. 87.2% (n = 232) of the participants accredited friends and family for the motivation to get the DPT3 vaccine, while 9.0% (n = 24), 1.1% (n = 3) and 2.6% (n = 7) gave credit to the university, social medial and government funded programs, respectively. Table 2 shows the relationship of various factors to the awareness of the participants. Participants who had received vaccination during childhood were more likely to be vaccinated (p-value = 0.030) and so were the participants whose families had vaccination routines (p-value = 0.019). Even though a higher percentage of fourth and final years were aware, the level of awareness did not appear to correlate with the year of study (p-value = 0.163).

**Table 2:** Factors affecting awareness regarding DPT3 vaccine DPT3: Diphtheria, pertussis and tetanus vaccine; N: Number

	Aware N (%)	Unaware N (%)	p-value
Gender			0.755
Female	162/202 (80.2)	40/202 (19.8)	
Male	66/84 (78.6)	18/84(21.4)	
Year of study?			0.163
Second and Third year	54/72 (75.0)	18/72 (25.0)	
Fourth and Fifth year	174/214 (81.3)	40/214 (18.7)	
Does your family have a vaccination routine?			0.019
Yes	154/184 (83.7)	30/184 (16.3)	
No	74/102 (72.5)	28/102 (27.5)	
Have you been vaccinated during childhood?			0.030
Yes	216/266 (81.2)	50/266 (18.8)	
No	12/20 (60.0)	8/20 (40.0)	

Half of the population marked ‘not being at risk’ as the reason for not getting vaccinated while the other half blamed ‘not knowing about the vaccine’, ‘not knowing where it was available’ or unavailability of the vaccine as their reasons. 86.0% (n = 246) of the population was unaware that the university had a vaccination program as a recommended part of the admission process. Our analysis concluded that among these medical students, 20.3% (n = 58) students were unaware, 69.2% (n = 198) participants were moderately aware while only 10.5% (n = 30) participants were well aware about DPT3 immunization.

## Discussion

Immunization averts an estimated two to three million deaths every year from diphtheria, tetanus, and pertussis [12]. By 2017, about 85% of infants worldwide received three doses of DPT3 vaccine, protecting them against the above-mentioned infectious diseases that can cause serious illness and disability or be fatal [13]. More than half of all children unvaccinated for DPT3 live in just five countries: Nigeria, India, Pakistan, Indonesia, and Ethiopia, reiterating the fact that these communicable diseases are now largely limited to the developing nations, possibly due to lack of efficient immunization programs [13, 14].

Adult coverage of immunization against diphtheria, tetanus, and particularly whooping cough caused by pertussis, is also required as immunity of the TdaP vaccine weans off to about 34% in two to four years after the primary doses [15]. Furthermore, adolescents and adults with waning immunity, especially immediate family members, are responsible for 76%-83% of pertussis transmission to infants [16]. Consequently, the Advisory Committee on Immunization Practices (ACIP), the national decision-making body for vaccine use in the United States, recommended in June 2005 that all adolescents routinely receive a single dose of TdaP to replace the next booster dose of Td, and later, the same recommendation was made for all adults [17, 18]. However, an American study found out that in spite of ACIP recommendation of DPT3 for adults, the percentage of adults in America who received any tetanus toxoid-containing vaccination during the past 10 years was 62.2% overall for aged ≥19 years, 62.6% for adults aged 19-49 years, 64.7% for adults aged 50-64 years, and 57.7% for adults aged ≥65 [19]. Since adult coverage of immunization against diphtheria, tetanus, and pertussis is especially important for healthcare workers, immunocompromised patients or people in close proximity of infected individuals, in February 2006, ACIP recommended TdaP for health care personnel as a priority group [18].

Our study focused on medical students in their clinical years exposed to a hospital environment on a daily basis. We assessed their knowledge about immunization against diphtheria, pertussis and tetanus and various aspects of its disease, along with their status regarding TdaP boosters. 93% of our participants reported to be vaccinated against DPT3 during childhood, while less than half of our participants reported getting adult boosters, which is comparable to an Italian study inquiring vaccination coverage among health care workers, in which coverage of Td boosters was found to be 15.5% [20]. In an analysis of sero-positivity against pertussis in Spain, only 31.2% were found to be seropositive for pertussis where 3.3% of those reports could be attributed to a past infection [21]. Furthermore, a study done on Greek nursing students, also showed insufficient vaccination rates, with only 74.6% of the participants reported to have received the DPT3 vaccine [22]. In the USA, overall Tdap vaccination of HCP aged ≥19 years reported in 2014 was only 42.1%. Moreover, among HCP aged ≥19 years with direct patient care responsibilities, Tdap vaccination coverage was 47.5 [19]. This goes on to show that there is a large population of medical students and HCPs which is under immunized and this could have severe repercussions, not only for them but also for others by possibly contributing to the spread of these preventable diseases.

In our study, only 10.5% of our participants could be regarded as well-aware about all educational aspects of DPT3 immunization. These statistics regarding knowledge appear to be greatly insufficient, especially as medical students have a greater burden of responsibility to keep themselves abreast with up-to-date immunization practices and recommendations for HCPs, for their welfare as well as their patients. In a study conducted among French students by Mellon et al., knowledge regarding TdaP vaccination was found to be grossly insufficient with only 36% of participants reporting the correct interval between two doses of Td boosters [23]. Another study in Canada reported just 63.2% correctly answered questions regarding the TdaP vaccine by HCPs [24]. This lack of knowledge also contributes to the ‘vaccine hesitancy’ among HCPs when recommending them to their patients, thereby possibly adding to the burden of these diseases.

ACIP has been clear on the importance of DPT3 immunization for health care professionals prioritizing it for various reasons [18]. The group of people that also need to be vigilant about this aspect of self-immunization include medical students that are exposed to hospitals and patients in their clinical years. Also, more because these health care students will eventually become practicing HCPs whose knowledge regrading vaccines should be adequate. A study by Kubli et al. emphasized the impact of educating health care students about immunization on their perceived level of importance regarding vaccines, the value of which cannot be overstated [25]. Loulergu and Launay also propose that health care students be subjected to the same immunization requirements as HCPs, as their vaccination status, knowledge and attitude regarding immunization practices is evidently unsatisfactory, corroborating our findings [26].

Majority of universities worldwide grant admissions to students only upon proof of adequate immunization, which includes TdaP coverage. According to an American survey, tetanus, diphtheria, and acellular pertussis vaccination was required by 66%, 70%, and 75% of nursing, MD (Doctor of Medicine)-granting, and DO (Doctor of Osteopathic Medicine)-granting schools in the US, respectively [27]. In our study, 86% of students were unaware that their university has a vaccination program as a recommended part of the admission process, despite the fact that the university, among few others, also requires attestation of DPT3 vaccination and boosters received by the applicants before they sit for the entrance exams. Thus, medical students are in reality not immunized appropriately as indicated by our study, which is due to failure of rigid implementation and verification system by the university, to screen unimmunized individuals, as well as failure in provision of vaccines and their cost.

In Pakistan, the main obstacle in coverage of DPT3 among health care professionals is the lack of implementation of said vaccination in their respective institutes, non-availability and unaffordability. In Pakistan, Extended Program of Immunization, a federally administered program, was established in 1978 with the purpose of nation-wide immunization of children under five years of age against preventable diseases such as tuberculosis, poliomyelitis, diphtheria, pertussis, tetanus and measles with WHO-recommended vaccines [28]. However, no initiative exists so far for the immunization of health care professionals in Pakistan. Such an initiative in Pakistan could vastly improve the immunization status of our HCPs and medical students [29].

Moreover, there is a scarcely any recent data on the vaccination coverage in Pakistan regarding any vaccine-prevented disease. This is another barrier to improving vaccination coverage of DPT3, among other vaccines, and successful eradication of these disease. The study in hand, to our knowledge, is a first of its kind, assessing vaccination coverage and its knowledge among medical students. Hopefully, this study adds to the pool of data regarding immunization and is a step forward in this much needed direction towards nationwide immunization and eradication of these diseases. Nevertheless, combined efforts from the government, HCPs and even medical students are required to achieve this goal.

## Conclusions

This analysis concludes that medical students in a major public-sector university of Karachi, currently enrolled in clinical years of study and hence exposed to a hospital setting regularly, have inadequate knowledge about diphtheria-pertussis-tetanus along with the role of its vaccination in adults. Vaccination coverage among these students also appears to be unsatisfactory, which can put them at risk of contracting these transmissible diseases in the high-risk environment of a hospital, as well as endanger patients that these unimmunized individuals come in contact with. Institutes are required to encourage and verify their student’s adherence to vaccination requirements.

## References

[1] World Health Organization (WHO (2019). World Health Organization (WHO): Tetanus. Immunization, Vaccination and Biologicals.

[2] Afshar M, Raju M, Ansell D, Bleck TP (2011). Narrative review: tetanus—a health threat after natural disasters in developing countries. Ann Intern Med.

[3] Fawibe AE (2010). The pattern and outcome of adult tetanus at a sub-urban tertiary hospital in Nigeria. J Coll Physicians Surg Pak.

[4] Zafar F, Rasheed J, Ghaffar HA (2012). Neonatal tetanus. Professional Med J.

[5] Liang JL, Tiwari T, Moro P, Messonnier NE, Reingold A, Sawyer M, Clark TA (2018). Prevention of pertussis, tetanus, and diphtheria with vaccines in the United States: recommendations of the advisory committee on immunization practices (ACIP). MMWR Recomm Rep.

[6] Baxter R, Bartlett J, Rowhani-Rahbar A, Fireman B, Klein NP (2013). Effectiveness of pertussis vaccines for adolescents and adults: case-control study. BMJ.

[7] Cherry JD (2012). Epidemic pertussis in 2012—the resurgence of a vaccine-preventable disease. N Engl J Med.

[8] (2019). World Health Organization (WHO): Diphtheria. http://www.who.int/immunization/monitoring_surveillance/burden/diphtheria/en/.

[9] (2019). Vaccine information statement: diphtheria, tetanus and pertussis (DTaP). Centre of Disease Control and Prevention (CDC). VIS.

[10] Choi JH, Choo EJ, Huh A (2010). Immunogenicity and safety of diphtheria-tetanus vaccine in adults. J Korean Med Sci.

[11] (2019). World Health Organization (WHO): Pertussis. http://www.who.int/immunization/diseases/pertussis/en/.

[12] (2019). World Health Organization (WHO): 10 facts on immunization. https://www.who.int/features/factfiles/immunization/en/.

[13] (2019). World Health Organization (WHO): immunization coverage. Immunization coverage factsheet.

[14] (2018). Vaccination and immunization statistics- UNICEF data. https://data.unicef.org/topic/child-health/immunization/.

[15] Acosta AM, DeBolt C, Tasslimi A (2015). Tdap vaccine effectiveness in adolescents during the 2012 Washington state pertussis epidemic. Pediatrics.

[16] Tan TQ, Gerbie MV (2010). Pertussis and patient safety: implementing Tdap vaccine recommendations in hospitals. Jt Comm J Qual Patient Saf.

[17] Broder KR, Cortese MM, Iskander JK (2006). Preventing tetanus, diphtheria, and pertussis among adolescents: use of tetanus toxoid, reduced diphtheria toxoid and acellular pertussis vaccines. Recommendations of the Advisory Committee on Immunization Practices (ACIP). MMWR Recomm Rep.

[18] Kretsinger K, Broder KR, Cortese MM (2006). Preventing tetanus, diphtheria, and pertussis among adults: use of tetanus toxoid, reduced diphtheria toxoid and acellular pertussis vaccine. Recommendations of the Advisory Committee on Immunization Practices (ACIP) and recommendation of ACIP, supported by the Healthcare Infection Control Practices Advisory Committee (HICPAC), for use of Tdap among health-care personnel. MMWR Recomm Rep.

[19] Williams WW, Lu P, O’Halloran A (2016). Surveillance of vaccination coverage among adult populations — United States, 2014. MMWR Surveill Summ.

[20] Fortunato F, Tafuri S, Cozza S, Martinelli D, Prato R (2015). Low vaccination coverage among Italian healthcare workers in 2013: contributing to the voluntary vs. mandatory vaccination debate. Hum Vaccin Immunother.

[21] Rodríguez de la Pinta ML, Lareo MIC, Torrell JMR (2016). Seroprevalence of pertussis amongst healthcare professionals in Spain. Vaccine.

[22] Noula M, Raftopoulos V, Gesouli E, Tsaprounis T, Deltsidou A (2008). Greek nursing students' immunization coverage: data from central continental Greece. Nurs Health Sci.

[23] Mellon G, Rigal L, Partouche H (2014). Vaccine knowledge in students in Paris, France, and surrounding regions. Can J Infect Dis Med Microbiol.

[24] MacDougall D, Halperin BA, MacKinnon-Cameron D, Li L, McNeil SA, Langley JM, Halperin SA (2015). Universal tetanus, diphtheria, acellular pertussis (Tdap) vaccination of adults: what Canadian health care providers know and need to know. Hum Vaccin Immunother.

[25] Kubli K, McBane S, Hirsch JD, Lorentz S (2017). Student pharmacists' perceptions of immunizations. Curr Pharm Teach Learn.

[26] Loulergue P, Launay O (2014). Vaccinations among medical and nursing students: coverage and opportunities. Vaccine.

[27] Libby TE, Lindley MC, Ahmed F, Stevenson J, Grabowsky M, Strikas RA (2014). Student vaccination requirements of U.S. health professional schools: a national survey. J Allied Health.

[28] (2019). World Health Organization (WHO). Expanded programme on immunization: Pakistan. http://www.emro.who.int/pak/programmes/expanded-programme-on-immunization.html.

[29] Nasir K, Khan KA, Kadri WM, Salim S, Tufail K, Sheikh HZ, Ali SA (2000). Hepatitis B vaccination among health care workers and students of a medical college. J Pak Med Assoc.

